# Hypersensitivity reaction to nedaplatin: A case report and literature review

**DOI:** 10.1097/MD.0000000000036690

**Published:** 2023-12-15

**Authors:** Kelun Gan, Daiyuan Ma

**Affiliations:** a North Sichuan Medical College, Nanchong, Sichuan Province, China; b Department of Oncology, Affiliated Hospital of North Sichuan Medical College, Nanchong, Sichuan Province, China.

**Keywords:** based anti, cancer drug, chemotherapy, hypersensitivity reactions, IgE, mediated allergic reactions, nedaplatin, platinum

## Abstract

**Rationale:**

Although rare, systemic hypersensitivity reactions to nedaplatin chemotherapy arise rapidly and can be life-threatening. The causes are unclear, and multiple potential mechanisms exist. Here, we report a case of systemic hypersensitivity reaction to nedaplatin and review the literature to establish a recommended protocol.

**Patient concerns:**

A 62-year-old man was being treated for squamous lung cancer with multiple metastases. On the first day of chemotherapy, 5 minutes after nedaplatin infusion, he developed panic, shortness of breath, and dyspnea with rapid heart rate, reduced oxygen saturation, and elevated blood pressure.

**Diagnoses:**

The symptoms indicated that the patient had developed a severe hypersensitivity reaction to nedaplatin, which could be life-threatening without immediate intervention.

**Intervention:**

Nedaplatin was discontinued, and he was treated with oxygen, ECG monitoring, finger pulse oximeter monitoring, 10 mg dexamethasone sodium phosphate injected intravenously, 20 mg diphenhydramine hydrochloride injected intramuscularly, and 40 mg methylprednisolone sodium succinate injected intravenously.

**Outcome:**

His allergic symptoms resolved, and once his vital signs stabilized, he was given 5 mg oral desloratadine once daily and 10 mg oral ebastine once daily to alleviate the effects of the allergic reaction. Once his vital signs remained stable without any special supportive treatment, he was discharged from the hospital. His chemotherapy regimen was discontinued, with no plan for a follow-up treatment due to the possibility of cross-allergic reactions between platinum-based drugs.

**Lessons:**

Clinical use of nedaplatin should be monitored and managed intensively for prevention and treatment of hypersensitivity reactions. Care should be taken to control the titration rate during infusion while closely monitoring vital signs. Clinical staff should be prepared to treat allergic symptoms as soon as they appear. The acute phase should involve immediate discontinuation of the drug; intravenous saline infusion for volume expansion; rapid assessment of circulation, airway, respiration, state of consciousness, and skin condition; and administration of oxygen, antihistamines, and epinephrine as appropriate for anaphylaxis. More randomized clinical trials are needed to elucidate appropriate preventative and management strategies to improve patient safety and support their successful completion of clinical treatment programs.

## 1. Introduction

Platinum drugs are among the most commonly used anti-cancer drugs. Because of their significant therapeutic effects and clear mechanisms of action, they are widely used in clinical practice, with 2- or 3-drug platinum-containing regimens having become the first-line chemotherapy regimen for many solid malignancies. First-generation platinum class cisplatin, second-generation platinum classes carboplatin and nedaplatin, and third-generation platinum classes oxaliplatin and lobaplatin have played critical roles in the treatment of malignant tumors.^[1]^ Although platinum drugs provide significant antitumor effects, as nonspecific cell-killing drugs, they carry the risk of significant adverse drug reactions, including myelosuppression, hepatotoxicity, nephrotoxicity, and neurotoxicity.^[2]^ Hypersensitivity reactions (HSRs) occur rapidly and can be life-threatening, creating a significant obstacle to successful implementation of chemotherapy. As the clinical use of platinum drugs has become more frequent, the incidence of HSRs has increased.^[3]^ Here, we report a case of systemic allergic reaction due to nedaplatin infusion and discuss the causes of platinum drug HSRs and their management in the context of the relevant literature.

## 2. Case report

The patient was a 62-year-old male with no history of food or drug allergy, heart disease, hypertension, or diabetes mellitus. On 11-08-2020, he was diagnosed with left lung squamous carcinoma with left hilar and mediastinal lymph node and brain metastases, with T4, N2, and M1b diseases (cT4N2M1b stage, IVa). He underwent 5 cycles of paclitaxel + cisplatin chemotherapy combined with tislelizumab immunotherapy and local radiotherapy, followed by tegafur + tislelizumab and vincristine + tislelizumab chemotherapy. On 07-26-2022, he was diagnosed with thoracic spine metastasis and was treated with radiotherapy. On 01-17-2023, he was evaluated as having progressive disease and was treated with tretinoin immunotherapy + (gemcitabine + cisplatin [GP]) (1400 mg gemcitabine on days 1 and 8 + 100 mg nedaplatin on days 1 and 2). On 04-03-2023, he was admitted to Affiliated Hospital of North Sichuan Medical College in Nanchong city, Sichuan Province, China, 2 + years after the diagnosis of left lung squamous carcinoma with brain metastasis, 2 + months after the diagnosis of disease progression during maintenance therapy after radiotherapy and 1 cycle of chemotherapy combined with immunotherapy. A physical examination recorded his body temperature as 36.3°C; pulse, 80.0 beats/min; respiration, 19.0 breaths/min; systolic blood pressure, 140.0 mm Hg; diastolic blood pressure, 101.0 mm Hg; height, 175.0 cm; weight, 75.0 kg; ECOG score, 1; KPS score, 90; and NRS score, 2~3. No obvious enlargement of the superficial lymph nodes was observed, and he presented with coarse breath sounds in both lungs but no obvious dry or wet rales. After admission, a chest CT scan indicated stable disease. He was diagnosed with left lung squamous carcinoma with left hilar, mediastinal lymph nodes, and bone and brain metastases (cT4N2M1c stage, IVb). Routine blood and biochemical tests revealed no significant abnormalities. On 04-04-2023, he did not complain of any special discomfort, and his vital signs were stable. On 04-05-2023, he began a new cycle of his GP treatment regimen (1400 mg gemcitabine on days 1 and 8 + 100 mg nedaplatin on days 1 and 2, every 3 weeks). Before chemotherapy, he was given 1.8 g glutathione injected intravenously for liver protection and metoclopramide hydrochloride injection, 10 mg metoclopramide hydrochloride injected intramuscularly and 100 mg dolasetron mesylate injected intravenously to prevent nausea and vomiting, and 5 mg dexamethasone sodium phosphate injected intravenously to prevent allergy. On the first day of chemotherapy, he was given 100 ml sodium chloride injection + 1400 mg gemcitabine hydrochloride injected intravenously at an intravenous drip rate of 40 drops per minute, and did not complain of any special discomfort. However, after 5 minutes of receiving the nedaplatin infusion, he developed panic, shortness of breath, and dyspnea. He was immediately given oxygen, and ECG monitoring and finger pulse oxygen monitoring revealed a heart rate of 150 beats/min, oxygen saturation of 80%, and blood pressure of 200/110 mm Hg. He was given 10 mg dexamethasone sodium phosphate injected intravenously and 20 mg diphenhydramine hydrochloride injected intramuscularly to alleviate the symptoms of allergy. This alleviated his symptoms slightly, and his oxygen saturation improved to 90%. Then, he was given 40 mg methylprednisolone sodium succinate injected intravenously to treat his severe allergic reaction. With this treatment, his symptoms of panic, shortness of breath, and dyspnea significantly improved, and his blood pressure decreased to 150/80 mm Hg. On 04-06-2023, his vital signs had stabilized, and he was given 5 mg oral desloratadine once daily and 10 mg oral ebastine once daily to alleviate the effects of the allergic reaction. On 04-07-2023, his vital signs remained stable without any special supportive treatment, and his general condition was assessed as acceptable, so he was discharged from the hospital. His chemotherapy regimen was discontinued, with no plan for a follow-up treatment, because we considered the possibility of cross-allergic reactions between platinum-based drugs and the fact that chemotherapeutic agents other than platinum-based may be difficult to achieve efficacy.

## 3. Discussion

Nedaplatin is a second-generation platinum drug that was first approved in Japan in 1995. It is widely used as the first-line treatment for non-small cell lung cancer, cervical cancer, ovarian cancer, nasopharyngeal cancer, esophageal cancer, and other common malignancies.^[4–6]^ Compared with first-generation platinum-based cisplatin, nedaplatin causes a significantly lower incidence of adverse reactions, such as hematologic toxicity, nephrotoxicity, and gastrointestinal symptoms, and does not require hydration at the time of use.^[7,8]^ HSR to nedaplatin is an IgE-mediated tachyphylaxis type I reaction,^[9]^ in which the patient body is already in a sensitized state after a first exposure to the allergen, and the HSR occurs upon re-exposure. Kawarada Y. et al^[10]^ retrospectively analyzed 111 patients who were treated with nedaplatin monotherapy, of whom 8 had experienced nedaplatin-associated HSRs (7.2%), most of whom developed allergic symptoms within 5 minutes of drug administration. The mechanism for the development of nedaplatin-associated anaphylactic reactions is unclear, although potential risk factors include: 1. A history of exposure to a prior platinum-based treatment, such as carboplatin, especially when there is a history of prior carboplatin-induced anaphylactic reactions. 2. Reintroduction of nedaplatin after an interval of ≥ 6 months following exposure to a prior platinum-based chemotherapy.^[10]^ Potential mechanisms of occurrence that have been supported by recent studies include: 1. Basophil overexpression of the IgE receptor-1 fragment (FcεRI) has been correlated with nedaplatin-induced anaphylaxis that is closely associated with a previous carboplatin treatment or allergy. Patients who develop a carboplatin-associated allergy reportedly have higher levels of basophil expression of the IgE receptor-1 fragment (FcεRI) and express higher levels of FcεRI mRNA in peripheral blood, which is an IgE-dependent mechanism of FcεRI overexpression is involved in carboplatin-induced severe HSRs^[11]^ and may be present in patients with nedaplatin-associated HSRs. 2. Allergen-specific IgE, an antibody the body produces as part of the allergic response to a specific allergen, has been observed in cases of carboplatin and oxaliplatin-associated allergic reactions and may be related to the primary amine moiety these drugs contain.^[12]^ Nedaplatin also contains this primary amine moiety, so it has been speculated that the induction of this moiety to produce a specific IgE may contribute to the development of HSRs in the organism.

The incidence of systemic HSRs caused by nedaplatin is low, but its occurrence is rapid and life-threatening, so although it should be given high priority in clinical application, appropriate preventive measures should be taken. The indications should be strictly understood by clinical staff in accordance with the drug instructions and disease guidelines before administration. Patients should be asked about their previous history of allergic reactions and platinum drug use. Unlike other platinum-related HSRs that typically occur during the eighth to ninth chemotherapy cycle,^[13]^ a study by Kawarada Y. et al^[10]^ revealed that nedaplatin may be more likely to cause HSRs during the first 2 to 3 cycles of the chemotherapy course, suggesting that in the clinical use of nedaplatin, patients should receive stricter preventative care prior to undergoing the course and then be closely monitored. Due to the small sample size of this retrospective study, further studies, including retrospective studies and prospective clinical trials with larger sample sizes, are needed to validate this conclusion. Premedication with glucocorticoids and antihistamines can reduce the symptoms of mild to moderate HSRs but cannot prevent severe rapid type I HSRs.^[14]^ Skin tests can be performed before the infusion to predict the risk of HSRs, especially in patients who have experienced more than 1 allergic reaction. When there is no other available regimen than platinum-based chemotherapy, routine skin tests are recommended to reduce the risk of HSRs.^[13,15]^ Care should be taken to control the titration rate during nedaplatin infusion, and the patient vital signs, including heart rate, pulse, respiration, and blood pressure, should be strictly monitored. The most common symptoms of platinum drug-related HSRs, including nedaplatin-induced HSRs, are skin manifestations (pruritus, urticaria, facial flushing, angioedema, palmar erythema, erythematous flushing) and other symptoms such as fever and/or shivering chills; gastrointestinal manifestations (abdominal pain, diarrhea, nausea); respiratory manifestations (dyspnea, bronchospasm); and circulatory manifestations (altered heart rate and blood pressure).^[16]^ Symptoms appear rapidly and manifest significantly, so it is recommended that clinical staff improve the efficacy of their multidisciplinary rounds to identify and treat allergic symptoms as soon as they appear. The acute phase of the allergic attack should be treated with immediate discontinuation of the drug, followed by intravenous saline infusion for volume expansion; rapid assessment of the patient circulation, airway, respiration, state of consciousness, and skin condition; determination of the severity of the allergy; administration of oxygen as appropriate; intravenous administration of antihistamines such as diphenhydramine and glucocorticoids; and rapid administration of epinephrine in severe cases of anaphylaxis at a dosage of 0.1% (0.01 mg/kg for any age and up to maximum of 0.5 mg for adults) by intramuscular injection.^[17,18]^ In this case, the nedaplatin infusion was stopped immediately after the onset of the allergic reaction, and after the administration of 10 mg dexamethasone sodium phosphate injected intravenously, 20 mg diphenhydramine hydrochloride injected intramuscularly, and 40 mg methylprednisolone sodium succinate injected intravenously, the patient allergic symptoms resolved rapidly and his vital signs gradually returned to normal.

Cross-HSRs are known to occur between cisplatin, carboplatin, and oxaliplatin,^[12]^ but there have been few studies on cross-allergies occurring between nedaplatin and other platinum drugs. Therefore, it is not recommended to switch to another platinum drug without skin testing after nedaplatin causes an allergic reaction. Desensitization offers a new possibility for continuing the safe use of platinum drugs after a platinum-associated allergic reaction. The most widely accepted platinum drug desensitization treatment is a 12-step regimen using 3 dilution multiples (1:10,1:10,1:1), increased by 2- to 2.5-fold each time depending on the in vitro mechanism of IgE-mediated mast cell desensitization.^[19,20]^ The efficacy of this desensitization regimen has been demonstrated in carboplatin- and oxaliplatin-related anaphylactic reactions^[21,22]^; however, to the authors’ knowledge, there have been no clinical trials on the efficacy of desensitization therapy after nedaplatin-induced anaphylactic reactions.

In summary, the incidence of HSRs to nedaplatin has been increasing in recent years, making it imperative for clinical practitioners to avoid serious adverse outcomes by paying sufficient attention to the risk and occurrence of nedaplatin-induced HSRs when nedaplatin is implemented as part of a chemotherapy regimen. Among the above-mentioned preventive and treatment measures, skin testing and desensitization therapy are promising directions for future research on nedaplatin-induced HSRs, as their effectiveness has been supported by other platinum drug-related trials. We expect more clinical trials on nedaplatin-related HSRs to lead to improved prevention and treatment standards and the continuous improvement of platinum drug safety.

## Acknowledgments

We thank Medjaden Inc. for its assistance in the preparation of this manuscript.

## Author contributions

**Data curation:** Kelun Gan.

**Investigation:** Kelun Gan.

**Project administration:** Daiyuan Ma.

**Supervision:** Daiyuan Ma.

**Writing – original draft:** Kelun Gan.

**Writing – review & editing:** Daiyuan Ma.
